# Neuroanatomical asymmetries in nonhuman primates in the homologs to Broca's and Wernicke's areas: a mini-review

**DOI:** 10.1042/ETLS20210279

**Published:** 2022-09-08

**Authors:** William D. Hopkins

**Affiliations:** Department of Comparative Medicine, Michale E Keeling Center for Comparative Medicine and Research, M D Anderson Cancer Center, Bastrop, TX 78602, U.S.A.

**Keywords:** brain asymmetry, chimpanzees, comparative brain evolution, laterality

## Abstract

Population-level lateralization in structure and function is a fundamental measure of the human nervous system. To what extent nonhuman primates exhibit similar patterns of asymmetry remains a topic of considerable scientific interest. In this mini-review, a brief summary of findings on brain asymmetries in nonhuman primates in brain regions considered to the homolog's to Broca's and Wernicke's area are presented. Limitations of existing and directions for future studies are discussed in the context of facilitating comparative investigations in primates.

## Introduction

A fundamental feature of the human brain is lateralization, which is defined as left-right differences neuroanatomy and neurofunctions that underlie different sensory, motor and cognitive functions [1–4]. Asymmetries in the structure are often conceptualized and quantified in terms of unilateral differences between homologous brain regions following a set of operationally defined neuroanatomical landmarks. More recent advances in neuroimaging techniques and quantification methods that operate at the level of the voxel or vertices have allowed for expansive, whole brain analyses of asymmetry in gray matter, white matter, cortical thickness, surface area and in anatomical and functional connectivity [5–9]. In humans, these approaches have revealed more comprehensive results with respect to whole brain variation in lateralization in morphology and connectivity.

In the study of behavioral and brain lateralization, scientists typically distinguish between individual and population-level asymmetries. Specifically, at the individual level, left-right difference measures obtained from bilateral traits are computed for each subject. The sign and absolute value indicate the direction and magnitude of a given subjects laterality and these are often described as asymmetry quotient (AQ) scores.

Population-level asymmetry indicates whether the average AQ scores for a sample of subjects differs from zero or, relatedly, whether a significant proportion of subjects within a sample show the same directional bias based on the sign of the AQ value. Thus, like other types of research on asymmetry, different distributions of lateralization can be obtained within a population or sample including: (1) a normal distribution of AQs, (2) a bimodal distribution AQ scores and (3) a rightward or leftward skewed distribution of AQ scores. Normal and bimodal distributions of AQ scores will have mean values on or about zero and reflect no population-level bias but for different reasons. In contrast, skewed distributions reflect population-level biases (depending on sample size and consistently of the asymmetry within the sample).

In the context of brain and behavioral asymmetries in nonhuman primates, it has been known for quite some time that individual subjects show asymmetries in certain behaviors, such as hand preferences or on measures of neuroanatomical structures [10–13]. What has been less documented and, indeed a matter of much greater debate, is whether nonhuman primates exhibit population-level asymmetries and, if they do, whether they are comparable in direction to those observed in humans. Indeed, the question of homology in population-level behavioral asymmetries has received considerable attention as it pertains to handedness [14–20]. However, there has been less research and thereby less discussion on the existence of population-level asymmetries in neuroanatomy in nonhuman primates and their similarities (and differences) to those observed in humans [21].

**Figure 1.**
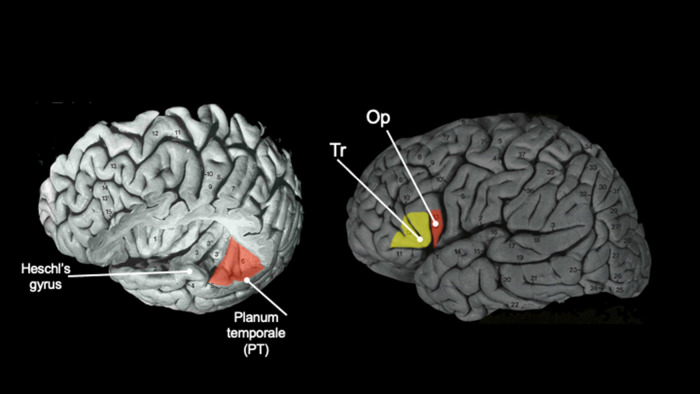
Morphological regions that comprise the planum temporale (left panel) and inferior frontal gyrus (right panel) of the human brain. OP = pars opercularis, Tr = pars triangularis.

In this review, I summarize recently published data on asymmetries in the nonhuman primate brain that have utilized more modern imaging technologies, with a specific emphasis on studies in chimpanzees. Notably, many early studies on brain asymmetries focused on linear measurements of the length of cortical sulci measured directly from postmortem brains [22–26], endocasts [27–29] or in a few cases magnetic resonance images [30–32]. In addition, several studies quantified asymmetries in the shape of the skull based on endocasts or related methods [33–35]. These collective findings have been summarized recently by Hopkins et al. [21]. In this mini-review, I will summarize more recent findings on brain asymmetries in nonhuman primates that have utilized region-of-interest methods. Furthermore, I will limit the review to studies that have quantified brain regions considered homologous to Broca's and Wernicke's areas in the human brain (see Figure 1). Specially, some of the first studies to describe lateralization in structure and function came from case studies of patients with unilateral damage to the left hemisphere inferior frontal gyrus (IFG) or posterior superior temporal gyrus (STG). In these cases, the patients exhibited deficits in language production and/or language comprehension whereas patients with damage to these same brain regions in the right hemisphere did not show any speech deficits. This led to the view that language functions are largely controlled by the left hemisphere, a finding largely confirmed over the next century in additional clinical cases and from experimental studies using a variety of neuropsychological test and measure employing functional imaging. Because many believe that language is uniquely humans, the historical and contemporary interest in brain asymmetries has largely focused on quantifying measures of brain regions or sulci that are homologous to Broca's and Wernicke's area.

### Planum temporale

**Figure 2.**
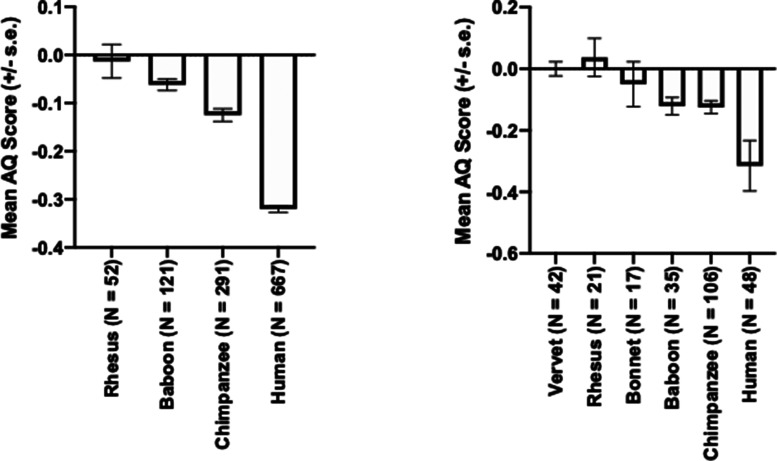
Mean AQ scores (±s.e.) for (**a**) PT surface area and (**b**) PT gray matter volume in different primate species.

Brodmann's area 22 (BA22) or Wernicke's area is found in the posterior STG [36,37]. The most reliable landmark to quantify this region is referred to as the planum temporale (PT), which is the bank of tissue that lies posterior the Heschl's gyrus (see Figure 1). Historically, scientists have quantified either the surface area or the gray matter volume of the PT in humans and consistently reported a significant leftward asymmetry (PT) (see Figure 2a and Table 1). With respect to nonhuman primates, in one of the first systematic comparative studies of PT asymmetry in chimpanzees, Gannon et al. [38] measured the surface area from a sample of 18 postmortem brains and reported a leftward asymmetry in 17 of the apes. In three subsequent studies, the PT surface area was measured either directly from MRI scans or from 3D reconstructions of the sulci in samples of chimpanzees and all three studies reported a significant population-level leftward asymmetry [39–42]. More recently, Marie et al. [43] quantified the PT in a sample of 96 adult baboons and reported a population-level leftward asymmetry. Following on from this report, Becker et al. [44] measured the PT in a sample of 35 newborn baboons (<3 months of age) and similarly found a leftward asymmetry. Lastly, in rhesus monkeys, no population-level bias has been found, a somewhat interesting result in light of the results reported in baboons, another Old World primate species.

**Table 1.** Planum temporale asymmetries in nonhuman primates.

	#L	#A	#R	Mean AQ	se	*t*
*Surface area*						
Chimpanzee	17	0	1	−0.574	0.132	−4.35**
Chimpanzee	159	21	43	−0.129	0.013	−9.55**
Chimpanzee	197	32	62	−0.104	0.013	−7.96**
Chimpanzee	7	1	2	NA		
Baboon	60	11	25	−0.070	0.013	−5.62**
Baboon	25	8	2	−0.058	0.011	−5.15**
Rhesus monkey	21	5	26	−0.013	0.035	−0.36
*Gray matter volume*						
Human	34	2	12	NA		
Chimpanzee	134	12	43	0.107	0.013	8.00**
Chimpanzee	198	25	68	−0.117	0.013	−8.98**
Baboon	24	3	7	−0.121	0.028	−4.20**
Rhesus monkey	10	3	8	0.040	0.042	0.95
Bonnett monkey	11	0	4	−0.052	0.056	
Vervet monkey	16	9	17	−0.002	0.023	
*Cytoarchitectonics*						
Humans	3	0	1	NA		
Chimpanzee (nn)	10	1	1	−0.401	0.141	−2.91*
Chimpanzee (nd)	8	0	4	−0.144	0.124	−1.17
Chimpanzee (vol)	9	0	3	−0.265	0.144	−1.89
Chimpanzee (np)	5	1	6	0.000	0.049	0.97
Rhesus (vol)	5	0	1	NA		

NA, not applicable or no data reported; se, standard error; n, neuron number; nd, neuron density; vol, volume.

In addition to the surface area, several authors have also measured the gray matter volume of the PT in nonhuman primates. Not surprisingly, the results for PT gray matter asymmetries are largely consistent with the surface area measures. This is because, assuming that cortical thickness in gray matter remains relatively consistent throughout the region, then the larger surface area will produce a larger gray matter volume. Thus, the leftward asymmetries observed for surface area in chimpanzees [41] and baboons [45] are similarly observed for gray matter volume (see Figure 2b and Table 1). In contrast, neither rhesus monkeys, bonnet monkeys or vervet monkeys show a population-level bias in gray matter volume for the PT [46] (see Figure 2b).

#### Cytoarchitectonics

There are remarkably few studies documenting asymmetries at the cellular level in BA22 in human and nonhuman primates (Table 1). In one of the only reported studies in humans, Galaburda et al. [47] reported a larger volume of BA22 in the left hemisphere of four postmortem brains. In chimpanzees, Spocter et al. [48] measured the volume, neuron number and neuron density of BA22 in 12 postmortem adult brains and reported a significant leftward asymmetry for each measure (see Table 1). In a follow up study, Spocter et al. [49] further quantified microstructural asymmetries in BA22 by measuring neuropil space and found no population-level bias. Finally, Gannon et al. [50] measured the volume of BA22 in 6 rhesus monkeys and found the 5 of the 6 individuals showed a leftward asymmetry.

### Inferior frontal gyrus (Broca's area homolog)

**Figure 3.**
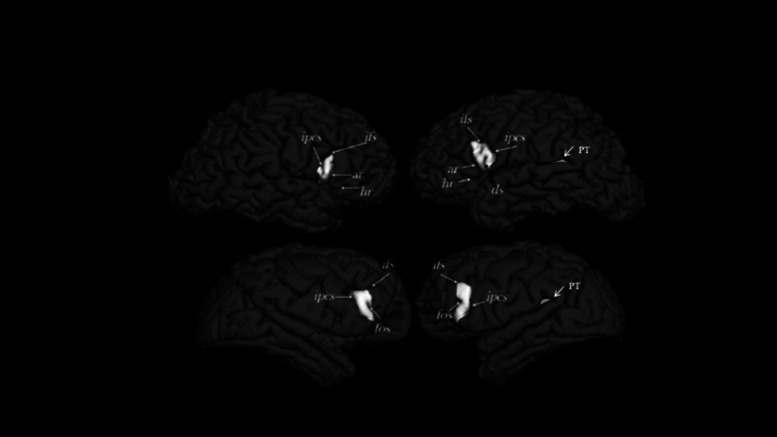
Sulci used to define the inferior frontal gyrus in humans (upper panel) and chimpanzees (lower panel). Ipcs = inferior precentral sulcus, ifs = inferior frontal sulcus, ar = ascending ramus, hr = horizontal ramus, ds = dimple sulcus, fos = fronto-orbital sulcus. From Keller et al. [58].

Comparative studies of the IFG in nonhuman primates are challenging because of differences in the sulcal landmarks the define the region (see Figure 3). Specifically, in humans, the IFG is broadly classified into the Pars opercularis (Op) and the Pars triangularis (Tr). The Pars opercularis is bordered superiorly by the inferior frontal sulcus (ifs), anteriorly by the anterior ascending ramus of the lateral fissure (aalf) and posteriorly by the inferior precentral sulcus (iprs). The Tr is bordered inferiorly by the horizonal ramus of the lateral fissure (half) and posteriorly by the ascending ramus, and together these rami form a ‘triangle’ shaped gyrus [51–53]. In chimpanzees, the sulci landmarks that define the Op are homologous; however, chimpanzees lack a horizontal ramus of the lateral fissure which precludes being able to define the Tr using sulci landmarks. In more distantly related Old and New World monkeys, the cortical folds are even less similar further complicating the measurement. Cytoarchitectonic studies in macaques and baboons have shown that BA44 and BA45, the constituent cellular components that derive the Op and Tr, respectively, are buried in the central portion of the arcuate sulcus [54,55]. Thus, some have suggested that measurement of the entire or portions of arcuate sulcus can be used as approximations to the measurement of Broca's area, at least in Old World monkeys (discussed below).

In three separate studies that have utilized region-of-interest methods, either no population-level or a slight rightward bias was reported in human subjects [56–58]. In a comparative study of the IFG gray matter volume in 30 human and 30 chimpanzees by Keller et al. [58], neither species showed a population-level bias (see Table 2). In one subsequent study measuring the total gray matter volume of the IFG in a much larger sample of chimpanzees (*n* = 189), Hopkins et al. [59] reported no population-level bias, consistent with the report by Keller et al. [58]. In contrast, studies by Cantalupo and Hopkins [60] reported a leftward asymmetry in the overall volume of the IFG in chimpanzees, bonobos and gorillas, albeit in much smaller samples of subjects.

**Table 2.** Asymmetries in Broca area homolog regions: volumetrics and cytoarchitectonics.

	#L	#A	#R	Mean AQ	se	*t*
Total volume						
Human	12	9	11	NA		
Human	NA			−0.082	0.035	−2.34*
Chimpanzee	30	4	23	−0.104	0.038	−2.71**
Chimpanzee	87	12	90	0.021	0.030	0.68
Bonobo	4	1	0	−0.097	0.054	−1.77
Gorilla	2	1	0	−0.068	0.023	−2.91
Gray matter volume						
Human	28	1	31	0.041	0.061	0.67
Human	12	0	18	−0.021	0.112	−0.001
Human	17	4	29	.084	0.074	1.13
Chimpanzee	12	0	18	0.052	0.066	0.79
Chimpanzee	87	12	90	0.021	0.030	0.68
Cytoarchitectonic						
*Human*						
BA44	10	0	0	NA		
BA45	6	0	4	NA		
*Chimpanzee*						
BA44 (nn)	5	1	6	0.029	0.169	0.17
BA44 (nd)	5	0	5	0.006	0.070	0.09
BA44 (vol)	5	1	6	0.075	0.144	0.60
BA45 (nn)	5	0	7	0.154	0.127	1.21
BA45(nd)	5	1	6	0.026	0.064	0.42
BA45 (vol)	4	0	8	0.245	0.127	1.94

NA, not applicable or no data reported; se, standard error.

**Figure 4.**
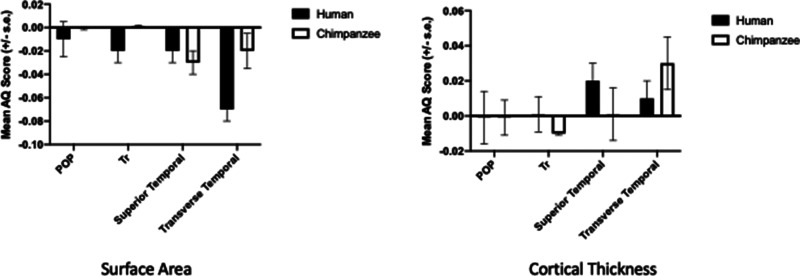
Mean AQ scores (±s.e.) for surface area and cortical thickness in the inferior frontal and posterior temporal gyri regions within the Desikan–Killany atlas maps applied to human and chimpanzee brains.

More recently, Xiang et al. [61] compared surface area and cortical thickness asymmetries between humans and chimpanzees. In this study, chimpanzee brains were aligned to a human brain template in the software program Freesurfer. Subsequently, the Desikan–Killany atlas maps, developed for use in humans, were subsequently applied to a sample of 77 chimpanzee brain scans and 91 scans of humans. With respect to the Op, Tr, STG and transverse temporal (TT) gyrus, the magnitude and direction of asymmetry were influenced by the different morphological measures (see Figure 4). For surface area, population-level leftward asymmetries were found for both chimpanzees in the superior temporal and TT gyri while no group level bias was found for either the POP or Tr. These results are largely consistent with the previous finding that have used traditional region-of-interest measures. For cortical thickness, no population-level asymmetries were found for either humans or chimpanzees for the Tr and POP regions. In humans, significant rightward asymmetries were found for the superior temporal but not the TT gyrus. In the chimpanzee, significant rightward asymmetries were found for the transverse but not STG.

## Cortical folding

### Surface area of frontal and temporal lobe sulci

**Figure 5.**
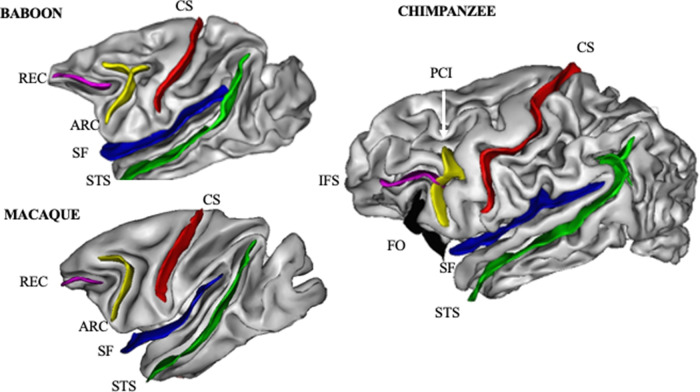
Lateral views of a macaque (rhesus monkey), baboon and chimpanzee brain with the frontal and temporal lobe sulci labeled for each species. REC = rectus, ARC = arcuate, IFS = inferior frontal sulcus, PCI = precentral inferior, CS = central sulcus, SF = sylvian fissure, and STS = superior temporal sulcus.

As noted above, early studies attempting to quantify brain asymmetries focused on measuring the length of select sulci in different primate species from either endocasts or directly from postmortem brains. In contrast with sulci length, recent studies incorporating MRI scans have quantified sulci surface area and the average depths by extracting cortical folds from the 3D scans using software programs such as Brainvisa (BV). To date, these approaches have been used to measure asymmetries in sulci within the temporal lobe including the sylvian fissure (SF) and superior temporal sulus (STS). Additional measures of asymmetry have been reported for frontal lobe sulci with the ventrolateral and inferior frontal gyri (see Figure 5).

With respect to the frontal lobe, Bogart et al. [62] measured the surface area and depth of the rectus (REC) and arcuate (ARC) in a sample of rhesus (*n* = 21) and bonnet (*n* = 28) macaque monkeys. Neither species showed a population bias for either folds (see Table 3). Becker et al. [63] measured the surface area and average depth of the ARC in 50 baboons and found no overall population-level bias (but see Discussion). The sulci that define the IFG include the fronto-orbital (FO), precentral inferior (PCI) and inferior frontal sulci (IFS). Using the software program BV, the surface areas and mean depth of the FO, PCI and IFS were quantified in the left and right hemispheres in 294 chimpanzees. For both surface area and depth, significant leftward asymmetries were found for the IFS and FO sulci but not PCI (see Table 3). In addition, the dorsal portion of the FO sulcus was reported to be significantly more often bifurcated in the left compared with the right hemisphere, a result consistent with a previous study describing the sulci patterns of the IFG in postmortem chimpanzee brains [64].

**Table 3.** Asymmetries in surface area of sulci within the inferior frontal gyrus and ventral lateral cortex.

	#L	#A	#R	Mean AQ	se	*t*
Sulci surface area						
*Chimpanzee*						
Fronto-orbital (FO)	170	52	96	−0.041	0.011	−3.58**
Precentral inferior (PCI)	136	40	142	−0.019	0.010	−1.94
Inferior frontal (IFS)	177	27	114	−0.142	0.031	−4.55**
Sylvian fissure (SF)	152	69	70	−0.032	0.006	−5.33**
Superior temporal sulcus (STS)	110	51	138	0.027	0.009	3.00**
*Baboon*						
Arcuate (ARC)	22	8	20	0.004	0.019	0.20
*Rhesus monkey*						
Arcuate (ARC)	6	3	12	0.080	0.045	1.76
Rectus (REC)	10	2	9	−0.012	0.123	−0.09
Sylvian fissure (SF)	12	3	6	0.060	0.043	−1.40
Superior temporal sulcus (STS)	8	0	13	0.016	0.023	0.68
*Bonnet Monkeys*						
Arcuate (ARC)	12	1	15	0.061	0.086	0.71
Rectus (REC)	12	2	14	0.122	0.106	1.15
Sylvian fissure (SF)	10	1	16	0.052	0.075	0.69
Superior temporal sulcus (STS)	7	1	20	0.092	0.033	2.28*

**Figure 6.**
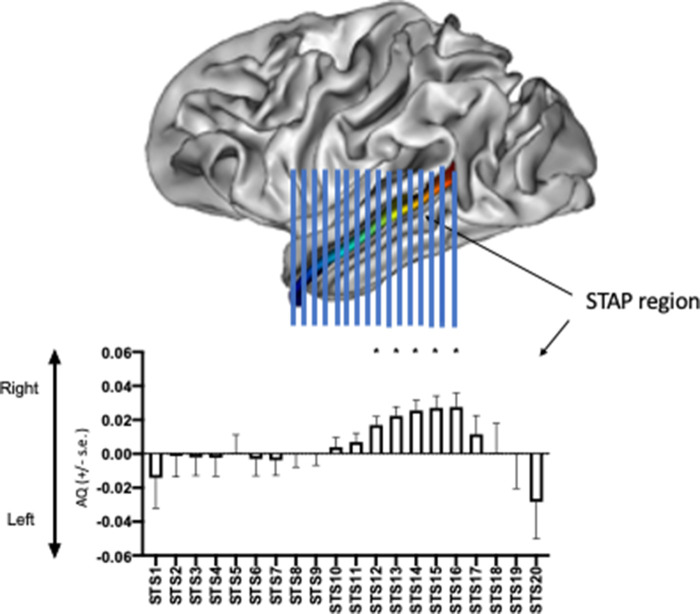
Mean AQ scores (±s.e.) for the STAP region in chimpanzees. The AQ values represent difference in the depth of the STS in 20 equally spaced anterior to posterior regions.

Regarding the temporal lobe sulci, chimpanzees showed a leftward asymmetry for the SF while neither the rhesus nor bonnet monkeys showed a population-level bias (see Table 3). For the STS, chimpanzees showed an overall rightward population-level bias as did the bonnet but not the rhesus monkeys. One interesting observation of the rightward STS asymmetry from the chimpanzee sample was the evidence that the rightward was particularly prominent in the area of the STS referred as the STAP (superior temporal asymmetric pit) [65]. In humans, significant and robust rightward asymmetries have been reported and it was hypothesized to be uniquely human (LeRoy et al. [66]). Contrary to this claim, chimpanzees showed a significant rightward asymmetry in the STAP region, though the magnitude of the bias was much smaller than data reported for human subjects (see Figure 6).

## Repeatability of asymmetries in chimpanzees

**Figure 7.**
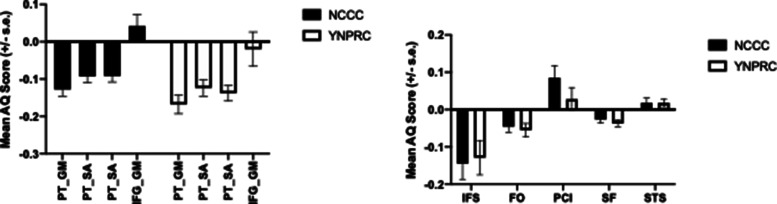
Mean AQ scores (±s.e.) for different measures of brain asymmetry in two independent samples of chimpanzees. NCCC = National Center for Chimpanzee Care (NCCC), YNPRC = Yerkes National Primate Research Center. Left panel represents PT surface area, gray matter volume and IFG gray matter volume. Right panel represents frontal and temporal lobe sulci surface area measures.

For the PT, IFG and sulci measures in the chimpanzees, the data were derived from two independent samples of captive apes. Specifically, one set of MRI data were obtained from chimpanzees housed at the National Center for Chimpanzee Care (NCCC) whereas the second cohort was collected in apes housed at the Yerkes National Primate Research Center (YNPRC). These two captive populations of apes were derived from distinct founder animals and there was no interbreeding that took place between individuals between the two populations. Thus, this arrangement provides an opportunity to compare the patterns of asymmetry between these two cohorts as a means of assessing their repeatability. As can be seen in Figure 7, patterns of asymmetry for the PT, IFG and the frontal and temporal lobe sulci were evident in both samples of chimpanzees suggesting the results are repeatable.

## Conclusion and parting thoughts

At the most basic level, and in contrast with many historical views, population-level asymmetries are evident in chimpanzees and, to a lesser extent, other nonhuman primate species. The most robust and consistent population-level asymmetry in chimpanzees and seemingly in baboons is the leftward bias evident in the PT. This finding has been reported in both species using multiple methods of analyses and at different levels of analysis including surface area and gray matter volume. Chimpanzees also show a leftward asymmetry in the volume and neuron density of the BA22 but there are no available data from baboons. It appears the rhesus monkeys may show a population-level leftward asymmetry at the cellular level for BA22 but this sample was relatively small and therefore the results should be interpreted cautiously, particularly in light of the lack of population-level bias in morphological measures from larger samples of subjects. Within the IFG, the evidence of population-level asymmetries seems less clear and appears to be high dependent on the type of measurement (i.e. sulci surface areas versus gray matter volume) and method of assessment. Performing comparative studies of brain asymmetry have several challenges and addressing them in future research would greatly benefit the field and theory. Three considerations for future research are discussed below.

### The confounding impact of handedness on studies of comparative brain asymmetry

As noted above, humans are predominantly and strongly right-handed whereas the evidence for population-level handedness in nonhuman primates is far less prevalent and, when evident, not nearly as strongly expressed [4,67,68]. For example, population-level right handedness has been reported in chimpanzees for multiple measures of hand use but the proportion of right-handed individuals approximates 65–70% compared with the 85–90% often reported in human subjects [18,19,69–71]. Because neuroanatomical asymmetries can be associated with handedness [72–77], the comparison of brain asymmetries between primate species is more complicated. Notably, in most studies of human subjects, the majority if not all of the subjects are right-handed whereas the handedness of subjects in many studies with nonhuman primates are either unknown or not reported. In instances where handedness was known in studies with nonhuman primates, significant brain-behavior associations have been reported in some but not all cases [78–84]. For instance, with respect to the ARC sulcus, Becker et al. [63] found that asymmetries in the depth of this fold were significantly more leftward in baboons that communicated gesturally more often with their right compared with the left hand. Similarly, Taglialatela et al. [85] found that chimpanzees that preferred to gesture with their right hand showed greater leftward asymmetries in the IFG compared with individuals that preferred their left hand or had no hand preference bias. In contrast, in both chimpanzees and baboons, hand preferences for bimanual actions were associated with asymmetries in the motor hand area of the precentral gyrus. These findings illustrate two important points. First, without considering or controlling for the handedness of subjects when comparing brain asymmetries between species, it is possible that the results might be biased due to an over representation of right- (or left-handed) individuals within one or more species. Second, at least in nonhuman primates, the type of measure of handedness appears to be associated with asymmetries in different cortical regions depending on their motor and communicative functions. This type of distinction in different dimensions of handedness is not typically considered when testing for association with brain asymmetries in humans.

### Brain size, effect size and sample size

Among primates, the human brain is ∼28% of its total volume at birth, which is much smaller compared with other primate species [86]. Additionally, compared with other primates, humans have a particularly long period of infant and juvenile periods of development. The relatively immature brain coupled with the longer period of infancy makes the human brain more susceptible to the influence of early social and environmental input on cortical development, including asymmetry [87–89]. Specifically, human and nonhuman primate newborns are not born into a symmetrical world. From birth (and arguably prior to birth), infants experience asymmetrical sensory and motor experiences, such as head orientation and nipple preferences by the infant and cradling biases by the primary caregiver which arguably can impact the development of both directional and absolute behavioral and brain asymmetries [90–100]. This is important for two reasons. First, depending on their consistency across individuals, the long-term impact of early asymmetrical input on the brain may result in more consistent and robust asymmetries in humans compared with other primate species. Thus, all other factors being equal, estimated effect sizes for the detection of population-level asymmetries will be greater in humans compared with other primate species. This, in turn, would be mean fewer subjects would be needed to detect population-level biases in humans compared with other primates. Samples sizes are already relatively small in many studies on asymmetries in nonhuman primates; therefore, it is possible that population-level asymmetries may be evident in nonhuman primate species for some measures but these studies are underpowered given the estimated smaller effect size.

### Whole brain approaches: templates (biased and unbiased)

Lastly, though not reviewed here, it is clear that voxel and surface-based whole brain approaches to the study of brain asymmetry in nonhuman primates are becoming increasingly evident in the literature [61,101–104]. There are many advantages to these approaches in the context of comparative studies of asymmetry in human and nonhuman primates. Notably, it allows for comparison in broadly defined brain regions between species without reliance on common sulci or related landmarks. That stated, there are some potential pitfalls that merit discussion. Notably, in whole brain approaches, the individual MRI scans through many registration steps are warped into a common stereotaxic space which is normally an ‘average’ or template brain for the species. The challenge is that inherent asymmetries in the template brain can exists which can potentially introduce biases in image registration and the measurement of individual asymmetries. Save the recent study by Xiang et al. [61], previous studies using voxel- or surfaced-based whole brain measures of asymmetries in nonhuman primates have not adopted preprocessing or analytic methods that assure that the template brains are indeed symmetrical.

### Summary

In sum, in many ways, the study of brain asymmetries in nonhuman primates is still in its infancy. Modern imaging technologies have allowed for scientists to increase the scope and complexity in the study of brain asymmetries at multiple levels of analysis. Compared with studies focused on postmortem tissue, modern imaging also allows for increased samples sizes which has historically and continues to be a significant limitation, particularly in light of the fact that the effect sizes may be smaller in the smaller brained nonhuman primates. At the same time, there remains a need for additional studies examining brain asymmetries at the cellular level of analysis, including in human brains. Lastly, like other aspects of cortical function in relation to complex behavior and cognition, lateralization in function likely operates through a series of connected nodes rather than discrete, isolated brain regions [105–110]. Thus, it is possible (and likely) that specific functions might be governed by a series of lateralized nodes that may be connected *within* and *between* hemispheres. Thus, studies may benefit by considering a comparative analysis of functional or anatomical connectivity rather than the whole brain or region-of-interest analyses.

## Summary

- Chimpanzees are an excellent model species for comparison to humans in the evolution of brain asymmetries.
- Individual and population-level leftward asymmetries are evident in the PT of chimpanzees at multiple levels of analysis. Similar leftward asymmetries appear evident in baboons but it less consistent in other Old World monkey species.
- Despite initial reports of leftward volumetric asymmetries in the homolog of Broca's area, evidence of population-level asymmetries is less clear cut in chimpanzees and humans.

## Abbreviations

AQ: asymmetry quotient
ARC: arcuate
BA22: Brodmann's area 22
BV: Brainvisa
FO: fronto-orbital
IFG: inferior frontal gyrus
Ifs: inferior frontal sulcus
PCI: precentral inferior
PT: planum temporale
REC: rectus
SF: sylvian fissure
STAP: superior temporal asymmetric pit
STG: superior temporal gyrus
STS: superior temporal sulus

## References

[1] Corballis, M.C. (1992) The Lopsided Brain: Evolution of the Generative Mind, Oxford University Press, New York, NY

[2] McManus, C. (2002) Right Hand, Left Hand: the Origins of Asymmetry in Brains, Bodies, Atoms, and Cultures, Weidenfeld & Nicolson, London

[3] Rogers, L.J., Vallortigara, G. and Andrew, R.J. (2013) Divided Brains: the Biology and Behaviour of Brain Asymmetries, Cambridge University Press, New York

[4] Ocklenburg, S. and Gunturkun, O. (2018) The Lateralized Brain: the Neuroscience and Evolution of Hemispheric Asymmetries, Academic Press, London 10.1080/1357650X.2018.1499749 30016906

[5] Saenger, V.M., Barrios, F.A., Martinez-Gudino, M.L. and Alcauter, S. (2012) Hemispheric asymmetries of functional connectivity and grey matter volume in the default mode network. Neuropsychologia 50, 1308–1315 10.1016/j.neuropsychologia.2012.02.014 22387608

[6] Toga, A.W. and Thompson, P.M. (2003) Mapping brain asymmetry. Nat. Rev. Neurosci. 4, 37–48 10.1038/nrn1009 12511860

[7] Luders, E., Gaser, C., Jancke, L. and Schlaug, G. (2004) A voxel-based approach to gray matter asymmetries. Neuroimage 22, 656–664 10.1016/j.neuroimage.2004.01.032 15193594

[8] Luders, E., Narr, K.L., Thompson, P.M., Rex, D.E., Jancke, L. and Toga, A.W. (2006) Hemispheric asymmetries in cortical thickness. Cereb Cortex 16, 1232–1238 10.1093/cercor/bhj064 16267139

[9] Buchel, C., Raedler, T., Sommer, M., Sach, M., Weiller, C. and Koch, M.A. (2004) White matter asymmetry in the human brain: a diffusion tensor MRI study. Cereb Cortex 14, 945–951 10.1093/cercor/bhh055 15115737

[10] LeMay, M. (1985) Asymmetries of the brains and skulls of nonhuman primates. In Cerebral Lateralization in Nonhuman Species (Glick, S.D., ed.), pp. 223–245, Academic Press, New York

[11] Warren, J.M. (1980) Handedness and laterality in humans and other animals. Physiol. Psychol. 8, 351–359 10.3758/BF03337470

[12] Ettlinger, G.F. (1988) Hand preference, ability and hemispheric specialization. How far are these factors related in the monkey? Cortex 24, 389–398 10.1016/S0010-9452(88)80002-9 3191723

[13] Finch, G. (1941) Chimpanzee handedness. Science 94, 117–118 10.1126/science.94.2431.117 17801500

[14] Macneilage, P.F., Studdertkennedy, M.G. and Lindblom, B. (1987) Primate handedness reconsidered. Behav. Brain Sci. 10, 247–263 10.1017/S0140525X00047695

[15] McGrew, W.C. and Marchant, L.F. (1997) On the other hand: current issues in and meta-analysis of the behavioral laterality of hand function in non-human primates. Am. J. Phys. Anthropol. 104, 201–232 10.1002/(SICI)1096-8644(1997)25+<201::AID-AJPA8>3.0.CO;2-69386827

[16] Hopkins, W.D. and Cantalupo, C. (2005) Individual and setting differences in the hand preferences of chimpanzees (*Pan troglodytes*): a critical analysis and some alternative explanations. Laterality 10, 65–80 10.1080/13576500342000301 15841824 PMC2147717

[17] Hopkins, W.D., Pika, S., Liebal, K., Bania, A., Meguerditchian, A., Gardner, M. et al, (2012) Handedness for manual gestures in great apes: a meta-analysis. In Current Developments in non-Human Primate Gesture Research (Pika, S. and Liebal, K., eds), pp. 93–111 John Benjamins Publishing, Amsterdam

[18] Hopkins, W.D. (2013) Comparing human and nonhuman primate handedness: challenges and a modest proposal for concensus. Dev. Psychobiol. 55, 621–636 10.1002/dev.21139 23913784 PMC4041077

[19] Hopkins, W.D., Gardner, M., Mingle, M., Reamer, L. and Schapiro, S.J. (2013) Within- and between-task consistency in hand use as a means of characterizing hand preferences in captive chimpanzees (*Pan troglodytes*). J. Comp. Psychol. 127, 380–391 10.1037/a0031071 23356440 PMC3842357

[20] Hopkins, W.D. (2006) Comparative and familial analysis of handedness in great apes. Psychol. Bull. 132, 538–559 10.1037/0033-2909.132.4.538 16822166 PMC2063575

[21] Hopkins, W.D., Misiura, M., Pope, S.M. and Latash, E.M. (2015) Behavioral and brain asymmetries in primates: a preliminary evaluation of two evolutionary hypotheses. Ann. N. Y. Acad. Sci. 1359, 65–83 10.1111/nyas.12936 26426409 PMC4715693

[22] Yeni-Komshian, G.H. and Benson, D.A. (1976) Anatomical study of cerebral asymmetry in the temporal lobe of humans, chimpanzees, and rhesus monkeys. Science 192, 387–389 10.1126/science.816005 816005

[23] Heilbronner, P.L. and Holloway, R.L. (1988) Anatomical brain asymmetries in New World and Old World monkeys. Stages of temporal lobe development in primate evolution. Am. J. Phys. Anthropol. 76, 39–48 10.1002/ajpa.1330760105 3136655

[24] Heilbronner, P.L. and Holloway, R.L. (1989) Anatomical brain asymmetry in monkeys: frontal, temporoparietal, and limbic cortex in Macaca. Am. J. Phys. Anthropol. 80, 203–211 10.1002/ajpa.1330800208 2801912

[25] Suwada, K. (2020) Cerebral sulcal asymmetry in macaque monkeys. Symmetry 12, 1–9 10.3390/sym12091509

[26] Imai, N., Sawada, K., Fukunishi, K., Sakata-Haga, H. and Fukui, Y. (2011) Sexual dimorphism of sulcal length asymmetry in the cerebrum of adult cynomolgus monkeys (*Macaca fascicularis*). Congenit. Anom. (Kyoto) 51, 161–166 10.1111/j.1741-4520.2011.00330.x 22103454

[27] Falk, D., Cheverud, J., Vannier, M.W. and Conroy, G.C. (1986) Advanced computer graphics technology reveals cortical asymmetry in endocasts of rhesus monkeys. Folia Primatol. (Basel) 46, 98–103 10.1159/000156242 3744188

[28] Falk, D., Hildebolt, C., Cheverud, J., Vannier, M., Helmkamp, R.C. and Konigsberg, L. (1990) Cortical asymmetries in the frontal lobe of rhesus monkeys (*Macaca mulatta*). Brain Res. 512, 40–45 10.1016/0006-8993(90)91167-F 2337807

[29] Cain, D.P. and Wada, J.A. (1979) An anatomical asymmetry in the baboon brain. Brain Behav. Evol. 16, 222–226 10.1159/000121838 114269

[30] Liu, S.T. and Phillips, K.A. (2009) Sylvian fissure asymmetry in capuchin monkeys (*Cebus apella*). Laterality 14, 217–227 10.1080/13576500802344404 18759195

[31] Hopkins, W.D., Pilcher, D.L. and MacGregor, L. (2000) Sylvian fissure length asymmetries in primates revisited: a comparative MRI study. Brain Behav. Evol. 56, 293–299 10.1159/000047213 11326134 PMC2018745

[32] Fears, S.C., Scheibel, K., Abaryan, Z., Lee, C., Service, S.K., Jorgensen, M.J. et al. (2011) Anatomic brain asymmetry in vervet monkeys. PLoS One 6, e28243 10.1371/journal.pone.0028243 22205941 PMC3244392

[33] Holloway, R.L. and Delacostelareymondie, M.C. (1982) Brain endocast asymmetry in pongids and hominids - some preliminary findings on the paleontology of cerebral-dominance. Am. J. Phys. Anthropol. 58, 101–110 10.1002/ajpa.1330580111 6812430

[34] Balzeau, A., Gilissen, E., Holloway, R.L., Prima, S. and Grimaud-Herve, D. (2014) Variations in size, shape and asymmetries of the third frontal convolution in hominids: paleoneurological implications for hominin evolution and the origin of language. J. Hum. Evol. 76, 116–128 10.1016/j.jhevol.2014.06.006 25042287

[35] Neubauer, S., Gunz, P. and Hublin, J.J. (2010) Endocranial shape changes during growth in chimpanzees and humans: a morphometric analysis of unique and shared aspects. J. Hum. Evol. 59, 555–566 10.1016/j.jhevol.2010.06.011 20727571

[36] Brodmann, K. (1909) Vergleichende Lokalisationslehre der Grobhirnrinde in ihren Prinzipien dargestellt auf Grund des Zellenbaues, Barth, Leipzig

[37] Wernicke, C. (1874) Der aphasische Symptomenkomplex, Cohn, Weigert, Breslau

[38] Gannon, P.J., Holloway, R.L., Broadfield, D.C. and Braun, A.R. (1998) Asymmetry of chimpanzee planum temporale: humanlike pattern of Wernicke's brain language area homolog. Science 279, 220–222 10.1126/science.279.5348.220 9422693

[39] Hopkins, W.D., Marino, L., Rilling, J.K. and MacGregor, L.A. (1998) Planum temporale asymmetries in great apes as revealed by magnetic resonance imaging (MRI). Neuroreport 9, 2913–2918 10.1097/00001756-199808240-00043 9760145

[40] Spocter, M.A., Sherwood, C.C., Schapiro, S.J. and Hopkins, W.D. (2020) Reproducibility of leftward planum temporale asymmetries in two genetically isolated populations of chimpanzees (*Pan troglodytes*). Proc. Biol. Sci. 287, 20201320 10.1098/rspb.2020.1320 32900313 PMC7542794

[41] Hopkins, W.D. and Nir, T. (2010) Planum temporale surface area and grey matter asymmetries in chimpanzees (*Pan troglodytes*): the effect of handedness and comparison within findings in humans. Behav. Brain Res. 208, 436–443 10.1016/j.bbr.2009.12.012 20035802 PMC2831152

[42] Gilissen, E., (2001) Structural symmetries and asymmetries in human and chimpanzee brains. In Evolutionary Anatomy of the Primate Cerebral Cortex (Falk, D. and Gibson, K.R., eds), pp. 187–215, Cambridge University, Cambridge

[43] Marie, D., Roth, M., Lacoste, R., Nazarian, B., Bertello, A., Anton, J.L. et al. (2018) Left brain asymmetry of the planum temporale in a nonhominid primate: redefining the origin of brain specialization for language. Cereb Cortex 28, 1808–1815 10.1093/cercor/bhx096 28431000

[44] Becker, Y., Sein, J., Velly, L., Giacomino, L., Renaud, L., Lacoste, R. et al. (2021) Early left-planum temporale asymmetry in newborn monkeys (*Papio anubis*): a longitudinal structural MRI study at two stages of development. Neuroimage 227, 117575 10.1016/j.neuroimage.2020.117575 33285330 PMC7896037

[45] Becker, Y., Phelipon, R., Sein, J., Velly, L., Renaud, L. and Meguerditchian, A. (2022) Planum temporale grey matter volume asymmetries in newborn monkeys (*Papio anubis*). Brain Struct. Funct. 227, 463–468 10.1007/s00429-021-02278-9 33937939

[46] Lyn, H., Pierre, P., Bennett, A.J., Fears, S., Woods, R. and Hopkins, W.D. (2011) Planum temporale grey matter asymmetries in chimpanzees (*Pan troglodytes*), vervet (*Chlorocebus aethiops sabaeus*), rhesus (*Macaca mulatta*) and bonnet (*Macaca radiata*) monkeys. Neuropsychologia 49, 2004–2012 10.1016/j.neuropsychologia.2011.03.030 21447349 PMC3151738

[47] Galaburda, A.M., Sanides, F. and Geschwind, N. (1978) Human brain. Cytoarchitectonic left-right asymmetries in the temporal speech region. Arch. Neurol. 35, 812–817 10.1001/archneur.1978.00500360036007 718483

[48] Spocter, M.A., Hopkins, W.D., Garrison, A.R., Stimpson, C.D., Erwin, J.M., Hof, P.R. et al. (2010) Wernicke's area homolog in chimpanzees (*Pan troglodytes*): probabilstic mapping, asymmetry and comparison with humans. Proc. R. Soc. B. Biol. Sci. 277, 2165–2174 10.1098/rspb.2010.0011

[49] Spocter, M.A., Hopkins, W.D., Barks, S.K., Bianchi, S., Hehmeyer, A.E., Anderson, S.M. et al. (2012) Neuropil distribution in the cerebral cortex differs between humans and chimpanzees. J. Comp. Neurol. 520, 2917–2929 10.1002/cne.23074 22350926 PMC3556724

[50] Gannon, P.J., Kheck, N. and Hof, P.R. (2008) Leftward interhemispheric asymmetry of macaque monkey temporal lobe language area homolog is evident at the cytoarchitectural, but not gross anatomic level. Brain Res. 1199, 62–73 10.1016/j.brainres.2007.12.041 18262172

[51] Keller, S.S., Crow, T., Foundas, A., Amunts, K. and Roberts, N. (2009) Broca's area: nomenclature, anatomy, typology and asymmetry. Brain Lang. 109, 29–48 10.1016/j.bandl.2008.11.005 19155059

[52] Keller, S.S., Highley, J.R., Garcia-Finana, M., Sluming, V., Rezaie, R. and Roberts, N. (2007) Sulcal variability, stereological measurement and asymmetry of Broca's area on MR images. J. Anat. 211, 534–555 10.1111/j.1469-7580.2007.00793.x 17727624 PMC2375829

[53] Petrides, M. (2019) Atlas of the Morphology of the Human Cerebral Cortex on the Average MNI Brain, Academic Press, New York

[54] Palomero-Gallagher, N. and Zilles, K. (2019) Differences in cytoarchitecture of Broca's region between human, ape and macaque brains. Cortex 118, 132–153 10.1016/j.cortex.2018.09.008 30333085

[55] Petrides, M., Cadoret, G. and Mackey, S. (2005) Orofacial somatomotor responses in the macaque monkey homologue of Broca's area. Nature 435, 1235–1238 10.1038/nature03628 15988526

[56] Knaus, T.A., Bollich, A.M., Corey, D.M., Lemen, L.C. and Foundas, A.L. (2006) Variability in perisylvian brain anatomy in healthy adults. Brain Lang. 97, 219–232 10.1016/j.bandl.2005.10.008 16300824

[57] Tomaiuolo, F., MacDonald, J.D., Caramanos, Z., Posner, G., Chiavaras, M., Evans, A.C. et al. (1999) Morphology, morphometry and probability mapping of the pars opercularis of the inferior frontal gyrus: an in vivo MRI analysis. Eur. J. Neurosci. 11, 3033–3046 10.1046/j.1460-9568.1999.00718.x 10510168

[58] Keller, S.S., Roberts, N. and Hopkins, W. (2009) A comparative magnetic resonance imaging study of the anatomy, variability, and asymmetry of Broca's area in the human and chimpanzee brain. J. Neurosci. 29, 14607–14616 10.1523/JNEUROSCI.2892-09.2009 19923293 PMC2797078

[59] Hopkins, W.D., Meguerditchian, A., Coulon, O., Misiura, M., Pope, S., Mareno, M.C. et al. (2017) Motor skill for tool-use is associated with asymmetries in Broca's area and the motor hand area of the precentral gyrus in chimpanzees (*Pan troglodytes*). Behav. Brain Res. 318, 71–81 10.1016/j.bbr.2016.10.048 27816558 PMC5459306

[60] Cantalupo, C. and Hopkins, W.D. (2001) Asymmetric Broca's area in great apes. Nature 414, 505 10.1038/35107134 11734839 PMC2043144

[61] Xiang, L., Crow, T.J., Hopkins, W.D. and Roberts, N. (2020) Comparison of surface area and cortical thickness asymmetry in the human and chimpanzee brain. Cereb Cortex, 1–15 10.1093/cercor/bhaa202 33026423 PMC10859246

[62] Bogart, S.L., Mangin, J.F., Schapiro, S.J., Reamer, L., Bennett, A.J., Pierre, P.J. et al. (2012) Cortical sulci asymmetries in chimpanzees and macaques: a new look at an old idea. Neuroimage 61, 533–541 10.1016/j.neuroimage.2012.03.082 22504765 PMC3358493

[63] Becker, Y., Claidiere, N., Margiotoudi, K., Marie, D., Roth, M., Nazarian, B. et al. (2022) Broca's cerebral asymmetry reflects gestural communication's lateralisation in monkeys (*Papio anubis*). eLife 11, e70521 10.7554/eLife.70521 35108197 PMC8846582

[64] Keller, S.S., Deppe, M., Herbin, M. and Gilissen, E. (2012) Variabilty and asymmetry of the sulcal contours defining Broca's area homologue in the chimpanzee brain. J. Comp. Neurol. 520, 1165–1180 10.1002/cne.22747 21826664

[65] Hopkins, W.D., Coulon, O., Meguerditchian, A., Staes, N., Sherwood, C.C., Schapiro, S.J. et al. (2022) Genetic determinants of individual variation in the superior temporal sulcus of chimpanzees (*Pan troglodytes*). Cerebral Cortex, in press 10.1093/cercor/bhac183 PMC9977371 35697647

[66] LeRoy, F., Cai, Q., Bogart, S.L., Dubois, J., Coulon, O., Monzalvo, K et al. (2015) New human-specific brain landmark: The depth asymmetry of superior temporal sulcus. PNAS 112, 1208–1213. 10.1073/pnas.1412389112 25583500 PMC4313811

[67] Porac, C. and Coren, S. (1981) Lateral Preferences and Human Behavior, Springer, New York

[68] Annett, M. (2002) Handedness and Brain Asymmetry: the Right Shift Theory, Psychology Press, Hove

[69] Hopkins, W.D., Russell, J.L., Cantalupo, C., Freeman, H. and Schapiro, S.J. (2005) Factors influencing the prevalence and handedness for throwing in captive chimpanzees (*Pan troglodytes*). J. Comp. Psychol. 119, 363–370 10.1037/0735-7036.119.4.363 16366769 PMC2680150

[70] Hopkins, W.D., Russell, J.L. and Schaeffer, J.A. (2012) The neural and cognitive correlates of aimed throwing in chimpanzees: a magnetic resonance image and behavioural study on a unique form of social tool use. Phil. Trans. R. Soc. B Biol. Sci. 367, 37–47 10.1098/rstb.2011.0195 PMC3223792 22106425

[71] Papademetriou, E., Sheu, C.F. and Michel, G.F. (2005) A meta-analysis of primate hand preferences for reaching and other hand-use measures. J. Comp. Psychol. 119, 33–48 10.1037/0735-7036.119.1.33 15740428

[72] Knecht, S., Drager, B., Deppe, M., Bobe, L., Lohmann, H., Floel, A. et al. (2000) Handedness and hemispheric language dominance in healthy humans. Brain 123, 2512–2518 10.1093/brain/123.12.2512 11099452

[73] Foundas, A.L., Hong, K., Leonard, C.M. and Heilman, K.M. (1998) Hand preference and magnetic resonance imaging asymmetries of the central sulcus. Neuropsychiatry Neuropsychol. Behav. Neurol. 11, 65–71 9652486

[74] Foundas, A.L., Leonard, C.M. and Hanna-Pladdy, B. (2002) Variability in the anatomy of the planum temporale and posterior ascending ramus: do right- and left handers differ? Brain Lang. 83, 403–424 10.1016/S0093-934X(02)00509-6 12468396

[75] Foundas, A.L., Leonard, C.M. and Heilman, K.M. (1995) Morphological cerebral asymmetries and handedness: the pars triangularis and planum temporale. Arch. Neurol. 52, 501–508 10.1001/archneur.1995.00540290091023 7733846

[76] Sha, Z., Pepe, A., Schijven, D., Carrion-Castillo, A., Roe, J.M., Westerhausen, R. et al. (2021) Handedness and its genetic influences are associated with structural asymmetries of the cerebral cortex in 31,864 individuals. Proc. Natl Acad. Sci. U.S.A. 118 10.1073/pnas.2113095118 PMC8617418 34785596

[77] Zetzsche, T., Meisenzahl, E.M., Preuss, U.W., Holder, J.J., Kathmann, N., Leinsinger, G. et al. (2001) In-vivo analysis of human planum temporale (PT): does the definition of PT borders influence the results with regard to cerebral asymmetry and correlation with handedness? Psychiatry Res. 107, 99–115 10.1016/S0925-4927(01)00087-7 11530276

[78] Phillips, K.A. and Sherwood, C.C. (2005) Primary motor cortex asymmetry is correlated with handedness in capuchin monkeys (*Cebus apella*). Behav. Neurosci. 119, 1701–1704 10.1037/0735-7044.119.6.1701 16420175

[79] Sherwood, C.C., Wahl, E., Erwin, J.M., Hof, P.R. and Hopkins, W.D. (2007) Histological asymmetries of primary motor cortex predicts handedness in chimpanzees (*Pan troglodytes*). J. Comp. Neurol. 503, 525–537 10.1002/cne.21399 17534947 PMC2680156

[80] Hopkins, W.D., Coulon, O. and Mangin, J.F. (2010) Observer-independent characterization of sulcal landmarks and depth asymmetry in the central sulcus of the chimpanzee brain. Neuroscience 171, 544–551 10.1016/j.neuroscience.2010.07.018 20813164 PMC2975865

[81] Hopkins, W.D. and Cantalupo, C. (2004) Handedness in chimpanzees is associated with asymmetries in the primary motor but not with homologous language areas. Behav. Neurosci. 118, 1176–1183 10.1037/0735-7044.118.6.1176 15598127 PMC2043153

[82] Meguerditchian, A., Gardner, M.J., Schapiro, S.J. and Hopkins, W.D. (2012) The sound of one-hand clapping: handedness and perisylvian neural correlates of a communicative gesture in chimpanzees. Proc. Biol. Sci. 279, 1959–1966 22217719 10.1098/rspb.2011.2485 PMC3311905

[83] Hamilton, C.R. and Vermeire, B.A. (1988) Complementary hemispheric specialization in monkeys. Science 242, 1691–1694 10.1126/science.3201258 3201258

[84] Hopkins, W.D., Taglialatela, J.P., Dunham, L. and Pierre, P. (2007) Behavioral and neuroanatomical correlates of white matter asymmetries in chimpanzees (*Pan troglodytes*). Eur. J. Neurosci. 25, 2565–2570 10.1111/j.1460-9568.2007.05502.x 17445252 PMC2654327

[85] Taglialatela, J.P., Cantalupo, C. and Hopkins, W.D. (2006) Gesture handedness predicts asymmetry in the chimpanzee inferior frontal gyrus. Neuroreport 17, 923–927 10.1097/01.wnr.0000221835.26093.5e 16738489 PMC2018746

[86] Leigh, S.R. (2004) Brain growth, life history, and cognition in primate and human evolution. Am. J. Primatol. 62, 139–164 10.1002/ajp.20012 15027089

[87] Gomez-Robles, A., Hopkins, W.D., Schapiro, S.J. and Sherwood, C.C. (2015) Relaxed genetic control of cortical organization in human brains compared with chimpanzees. Proc. Natl Acad. Sci. U.S.A. 112, 14799–14804 10.1073/pnas.1512646112 26627234 PMC4672807

[88] Gomez-Robles, A., Hopkins, W.D., Schapiro, S.J. and Sherwood, C.C. (2016) The heritability of chimpanzee and human brain asymmetry. Proc. Biol. Sci. 283, 20161319 28003442 10.1098/rspb.2016.1319 PMC5204159

[89] Gomez-Robles, A., Hopkins, W.D. and Sherwood, C.C. (2014) Modular structure facilitates mosaic evolution of the brain in chimpanzees and humans. Nat. Commun. 5, 4469 10.1038/ncomms5469 25047085 PMC4144426

[90] Hepper, P.G., Wells, D.L. and Lynch, C. (2005) Prenatal thumb sucking is related to postnatal handedness. Neuropsychologia 43, 313–315 10.1016/j.neuropsychologia.2004.08.009 15707608

[91] Previc, F.H. (1991) A general theory concerning the prenatal origins of cerebral lateralization in humans. Psychol. Rev. 98, 299–334 10.1037/0033-295X.98.3.299 1891521

[92] Michel, G.F., Campbell, J.M., Marcinowski, E.C., Nelson, E.L. Babik, I. (2016) Infant hand preference and the development of cognitive abilities. Front. Psychol. 7 10.3389/fpsyg.2016.00410 PMC4803747 27047431

[93] Hopkins, W.D. (2004) Laterality in maternal cradling and infant positional biases: implications for the development and evolution of hand preferences in nonhuman primates. Int. J. Primatol. 25, 1243–1265 10.1023/B:IJOP.0000043961.89133.3d 18049716 PMC2099253

[94] Regaiolli, B., Spiezio, C. and Hopkins, W.D. (2018) Asymmetries in mother-infant behaviour in Barbary macaques (*Macaca sylvanus*). PeerJ 6, e4736 10.7717/peerj.4736 PMC5947039 29761052

[95] Damerose, E. and Hopkins, W.D. (2002) A comparison of scan and focal sampling procedures in the assessment of laterality for maternal cradling and infant nipple preferences in olive baboons (*Papio anubis*). Anim. Behav. 63, 511–518 10.1006/anbe.2001.1931

[96] Hopkins, W.D. and De Lathouwers, M. (2006) Left nipple preferences in infant *Pan paniscus* and *P. troglodytes*. Int. J. Primatol. 27, 1653–1662 10.1007/s10764-006-9086-4 18185838 PMC2186093

[97] Jaffe, B.D., Evans, T.A., Howell, S., Westergaard, G.C., Snoy, P.J., Higley, J.D. (2006) Left versus right nipple preference in free-ranging infant rhesus macaques (*Macaca mulatta*). Dev. Psychobiol. 48, 266–272 10.1002/dev.20128 16568413

[98] Nishida, T. (1993) Left nipple suckling preference in wild chimpanzees. Ethol. Sociobiol. 14, 45–51 10.1016/0162-3095(93)90017-C

[99] Zhao, D., Gao, X., Li, B. and Watanabe, K. (2008) First wild evidence of neonate nipple preference and maternal cradling laterality in Old World monkeys: a preliminary study from *Rhinopithecus roxellana*. Behav. Process. 77, 364–368 10.1016/j.beproc.2007.10.004 18031955

[100] Harris, L.J. (2010) Side biases for holding and carrying infants: reports from the past and possible lessons for today. Laterality 15, 56–135 10.1080/13576500802584371 19296365

[101] Vickery, S., Hopkins, W.D., Sherwood, C.C., Schapiro, S.J., Latzman, R.D., Caspers, S. et al. (2020) Chimpanzee brain morphometry utilizing standardized MRI preprocessing and macroanatomical annotations. eLife 9, e60136 10.7554/eLife.60136 33226338 PMC7723405

[102] Hopkins, W.D., Taglialatela, J.P., Meguerditchian, A., Nir, T., Schenker, N.M. and Sherwood, C.C. (2008) Gray matter asymmetries in chimpanzees as revealed by voxel-based morphometry. Neuroimage 42, 491–497 10.1016/j.neuroimage.2008.05.014 18586523 PMC2569890

[103] Hopkins, W.D., Taglialatela, J.P., Nir, T., Schenker, N.M. and Sherwood, C.C. (2010) A voxel-based morphometry analysis of white matter asymmetries in chimpanzees (*Pan troglodytes*). Brain Behav. Evol. 76, 93–100 10.1159/000319010 20881357 PMC3202944

[104] Xia, J., Wang, F., Wu, Z., Wang, L., Zhang, C., Shen, D. et al. (2020) Mapping hemispheric asymmetries of the macaque cerebral cortex during early brain development. Hum. Brain Mapp. 41, 95–106 10.1002/hbm.24789 31532054 PMC7267900

[105] Raemaekers, M., Schellekens, W., Petridou, N. and Ramsey, N.F. (2018) Knowing left from right: asymmetric functional connectivity during resting state. Brain Struct. Funct. 223, 1909–1922 29299691 10.1007/s00429-017-1604-y PMC5884915

[106] Liu, H.S., Stufflebeam, S.M., Sepulcre, J., Hedden, T. and Buckner, R.L. (2009) Evidence from intrinsic activity that asymmetry of the human brain is controlled by multiple factors. Proc. Nat Acad. Sci. U.S.A. 106, 20499–20503 10.1073/pnas.0908073106 PMC2777963 19918055

[107] Joliot, M., Tzourio-Mazoyer, N. and Mazoyer, B. (2016) Intra-hemispheric intrinsic connectivity asymmetry and its relationships with handedness and language lateralization. Neuropsychologia 93, 437–447 10.1016/j.neuropsychologia.2016.03.013 26988116

[108] Yan, H., Zuo, X.N., Wang, D., Wang, J., Zhu, C., Milham, M.P., (2009) Hemispheric asymmetry in cognitive division of anterior cingulate cortex: a resting-state functional connectivity study. Neuroimage 47, 1579–1589 10.1016/j.neuroimage.2009.05.080 19501172

[109] Mechelli, A., Friston, K.J., Frackowiak, R.S. and Price, C.J. (2005) Structural covariance in the human cortex. J. Neurosci. 25, 8303–8310 10.1523/JNEUROSCI.0357-05.2005 16148238 PMC6725541

[110] Cheng, L., Zhang, Y., Li, G., Wang, J., Sherwood, C., Gong, G. et al. (2021) Connectional asymmetry of the inferior parietal lobule shapes hemispheric specialization in humans, chimpanzees, and rhesus macaques. eLife 10, e67600 10.7554/eLife.67600 34219649 PMC8257252

